# Anti-*Ehrlichia* properties of the essential oil of *Ageratum conyzoides* L. and its interaction with doxycycline

**DOI:** 10.1186/s13568-019-0780-y

**Published:** 2019-04-29

**Authors:** Carla Janaina Rebouças Marques do Rosário, Cláudia Quintino da Rocha, Daniel Moura de Aguiar, Cristian Alex Aquino Lima, Daniella Patrícia Brandão Silveira, José Antônio Costa Leite, Denise Fernandes Coutinho, Ferdinan Almeida Melo

**Affiliations:** 1 Laboratory of Immunodiagnosis, State University of Maranhão, São Luís, MA Brazil; 2 Laboratory of Advanced Studies in Phytomedicines, Department of Chemistry, Center for Exact Sciences and Technology, Federal University of Maranhão, São Luís, MA Brazil; 3 Laboratory of Virology and Rickettsioses, Faculty of Veterinary Medicine, Federal University of Mato Grosso, Cuiabá, MT Brazil; 4 Laboratory of Pharmacognosy II, Department of Pharmacy, Federal University of Maranhão, São Luís, MA Brazil

**Keywords:** Alternative treatment (alternative therapy), Mentrasto (*Ageratum conyzoides*, billygoat-weed), Association, DH82 cells, Precocene I

## Abstract

Canine Monocytic Ehrlichiosis (CME) is an infectious disease caused by the rickettsia organism *Ehrlichia canis* which is transmitted mainly the ixodid brown dog tick *Rhipicephalus sanguineus*. The prevalence of *E. canis* infection has been increasing in recent years. The World Health Organization has been warned about antibiotics resistance and one of the way to prevent this situation is found new compound with this property. Doxycycline is the treatment of choice for this tick-borne disease. Adverse effects are noted in dogs that are sensitive to this drug. Antibiotic resistance may also occur. The present study aimed to evaluate the anti-*Ehrlichia* properties of the essential oil of the aerial parts of *Ageratum conyzoides* L. in infected DH82 cells, as well as its anti-*Ehrlichia* activity associated with doxycycline using the checkerboard assay. *A. conyzoides* is a native plant from northeast Brazil with many reports of ethnopharmacological applications. The essential oil of *A. conyzoides* was extracted from the aerial parts of the plant using the hydrodistillation method. *E. canis*-infected DH82 cells were cultured in DMEM (Dulbecco’s Modified Eagle Medium), maintained at 37 °C and 5% CO_2_, and standardized at a 70% infection rate for the initiation of treatment protocols. The tests were first carried out with the aim of defining the IC_50_. The combined effect of doxycycline and *A. conyzoides* essential oil was then determined using the checkerboard dilution technique (checkerboard method) in which the IC_50_ was 200 µg/mL. The doxycycline reduction index from the combined effect was 4.90 times resulting in a synergistic effect. To the authors’ knowledge, this is the first alternative treatment (alternative therapy) based on bioactive molecules that have antibacterial activity against *E. canis*.

## Introduction

CME (Canine Monocytic Ehrlichiosis) is an infectious disease caused by *Ehrlichia canis,* a rickettsial organism of the family Anaplasmataceae which is transmitted mainly by the ixodid brown dog tick *Rhipicephalus sanguineus* (Stich et al. 2008).

*Ehrlichia* infection in dogs has been diagnosed worldwide but is particularly prevalent in tropical and subtropical regions. In Brazil, the prevalence of this diseases in dogs presented to hospitals and veterinary clinics varies between 4 and 76%. In addition, it is an important zoonosis (Makino et al. 2016).

The clinical signs, the histopathological findings, and even the persistence of the infection during the course of CME are directly related to the immune response developed by the host (Villaescusa et al. 2012).

The disease consists of three stages: acute, subclinical, and chronic. In the acute form of the disease, the clinical signs are nonspecific and include fever, ocular and nasal discharge, anorexia, depression, weight loss, dyspnea, lymphadenopathy, vasculitis, and neurological, muscular, ocular and articular manifestations (polyarthritis). Following the acute phase of the illness, spontaneous healing may occur or the animal develops subclinical disease. Immunocompetent dogs usually eliminate the bacteria whereas dogs with an insufficient immune response tend to develop the chronic form of the disease which can be fatal if not properly treated (Dagnone et al. 2001).

The standard treatment protocols currently used for CME advocate the use of some antimicrobials that may cause adverse effects as well as antibiotic resistance. Among these antimicrobials, the class of tetracyclines and amphenicois are considered the treatment of choice against *Ehrlichia* (Perez et al. 2006).

Essential oils (EO) are complex heterogeneous mixtures consisting of a great diversity of hydrophobic molecules which diffuse easily through all cell membranes showing advantages in interactions with intracellular targets. Properties of essential oils include low density and rapid diffusion through cell membranes due to its liposolubility. In addition, it can also improve the intracellular insertion of the active components into their targets (Santos et al. 2012).

The World Health Organization has been warned about antibiotics resistance and one of the way to prevent this situation is found new compound with this property (Organização Mundial de Saúde 2018). Thus plants represents important source of new drugs. *Ageratum conyzoides* L. (Asteraceae) is an annual herbaceous plant very common in the Brazilian northeast with many traditional medicinal and has bioactivity like antimicrobial (Kissmann et al. 1999).

*Ageratum conyzoides* L. (popular names: billygoat-weed, mentrasto) is a native plant from northeast Brazil with many reports of ethnopharmacological applications (Zucchi et al. 2013). It has anti-inflammatory (Mello et al. 2016) and antiparasitic activities (Narender et al. 2004) among other properties that have been reported in the literature. The present study aimed to evaluate the anti-*Ehrlichia* properties of the essential oil of *Ageratum conyzoides* L. on DH82 cells in view of the comprehensiveness of this species in northeast Brazil and its proven antimicrobial and antiprotozoal action. In addition, the anti-ehrlichial activity of this hydrophobic liquid in association with doxycycline was assessed using the checkerboard assay.

## Materials and methods

### Essential oil extraction

In this study, we used 200 g of fresh aerial parts of *A. conyzoides* collected in the early morning of July of 2017 at the Horto Berta Langes de Morretes, Federal University of Maranhão (UFMA), located in the municipality of São Luís, State of Maranhão (MA), northeast Brazil, Lat. 2°33′13.5″S 44°18′20.8″W. Samples were taxonomically identified and desiccated (voucher) specimens were deposited in the Herbarium of Maranhão—UFMA under the ID number Nº. 9.099. The essential oil of *A. conyzoides* was extracted from by hydrodistillation in a clevenger apparatus coupled to a Quimis ultrathermostatic bath with a temperature less than 12 °C. The aerial parts of the plant were crushed and placed in a conical flask added with ultrapure distilled water. After 2 h of distillation, the oil was removed from the water surface, centrifuged, and carefully separated from the water added with anhydrous sodium sulfate (JT Baker Chemical Co.), stored in an amber glass ampoule, hermetically sealed, and then stored in a cooled 4 °C for further analysis (Coutinho et al. 2007).

The access was registered under the ID number ADBBA07 in the National System of Management of Genetic Heritage and Associated Traditional Knowledge according to art. 41 of Decree No. 8.772/2016 of the Ministry of the Environment in Brazil.

### Characterization of the essential oil by GC/MS

The chemical composition of the essential oil was analyzed by gas phase chromatography/mass spectrometry (GC/MS) with the injection of 1 μL (Auto Injector AOC-20i) in a GCMS-QP 2010 Ultra (Shimadzu) equipped with a Rtx-5MS silica capillary column (Restek, USA) 30 m long × 0.25 mm inner diameter coated with 5%—diphenyl/95%—dimethyl-polysiloxane (0.25 μm film thickness).

The temperature of the GC oven was programmed from 60 to 240 °C at 3 °C/min, injector (1:20 split). Transfer line and ionization chamber temperatures were 250 °C, 250 °C, and 200 °C, respectively. Helium was used as the entrainment gas at a rate of 1 μL/min.

The mass spectra were obtained by electronic impact at 70 eV with automatic scans in the mass range between 35 and 400 m/z at 0.30 scans/s.

The identification of the components was based on the time and linear retention index (series of C8–C28 n-alkanes) and on the interpretation and comparison of the mass spectra obtained from the libraries (Adams 2012; NIST 2011).

### Microorganism

DH82 cells (Canine Histiocyte: ATCC No. CRL-10389) infected with 35th passage of the Cuiabá #1 strain of *E. canis* were cultured in Dulbecco’s Modified Eagle’s (DMEM) medium (Sigma Chemical Co., St. Louis), MO, USA) supplemented with 5% fetal calf serum (HyClone Laboratories, Logan, Utah, USA) and maintained in a 25 cm^2^ culture bottle at 37 °C with 5% CO_2_ as recommended by Aguiar et al. (2007). *E. canis* infection rate was determined by examining (screening) smears from a monolayer cell stained by the Diff-Quik Kit (Laborclin, Pinhais, PR, Brazil) under the light microscope.

When an rickettsial infection rate of 70% was detected using this method, the cells were resuspended with the same effect and the cell suspension was centrifuged at 4000*g* for 5 min. The experiments were run on 24-well culture plates at 37 °C with 5% CO_2_. The bacterial rate was standardized as 3000 cells per well and 70% of the cells infected with the rickettsia.

The access was registered under number A9463BB in the National System of Management of Genetic Heritage and Associated Traditional Knowledge according to art. 41 of Decree No. 8.772/2016 of the Brazilian Ministry of the Environment.

### Biological assay

The assays were performed in triplicate at concentrations of 25, 50, 100, 200, 300, 400, and 500 µg/mL of the essential oil of *A. conyzoides* L. plus 1% Dimethylsulfoxide-DMSO (Merck Chemical Co.) in order to solubilize the sample and at concentrations of 0.25, 0.50, 0.75, 1.0, 1.5 µg/mL of the doxycycline plus 1% Dimethylsulfoxide-DMSO (Merck Chemical Co.). Analyses were performed at 18 h and 36 h after addition of the treatments to the medium. The analyses consisted of counting the percentage of infected cells on Diff-Quik (Laborclin, Pinhais, PR, Brazil) stained cell monolayer smear preparations examined under the light microscope (Aguiar et al. 2007).

The experiments were performed according to the method published by Chou (2006). The treatments were tested at constant ratios of equipotent concentrations, ranging from 0.0625-, 0.125-, 0.25-, 0.5-, 1-fold of their respective IC_50_ value that was determined for each experiment. The synergism, antagonism, or additive effect of each of the combinations was assessed by calculating the combination index value (CI). According to Chou (2006) and Chou (1976):

1 $$ \frac{{d_{1} }}{{d_{1} x}} + \frac{{d_{2} }}{{d_{2} x}} $$

in which D_1_ and D_2_ are the doses of doxycycline (1) and EO (2), respectively, that are responsible for an effect *x* in combination whereas D_1_*x* and D_2_*x* are the doses of 1 and 2, respectively, that are responsible for the same effect individually. If CI < 1, the treatments have a synergistic effect; if CI > 1, they are antagonistic; if CI = 1, an additive effect is observed. A normalised isobologram was created by plotting the normalised concentrations $\frac{{d_{1} }}{{d_{1} x}}$ of 1 and $\frac{{d_{2} }}{{d_{2} x}}$ of 2 on the y- and x-axis, respectively, in which the denominators represent the respective doses of c 1 and 2 alone reducing antibacterial load by *x*%, and the numerators represent the respective doses of 1 and 2 reducing bacterial load by *x*% in combination. The normalised concentrations were calculated considering that

2 $$ \frac{{D_{1} }}{{D_{1} x}} = \frac{{D_{1} }}{{Dm_{1} \cdot \left( {\frac{fa}{1 - fa}} \right)^{{\frac{1}{{m_{1} }}^{{}} }} }} $$

in which $D_{{m_{1} }}$ is the IC_50_ of 1 in vitro, *f*_*a*_ is the fraction affected (or (% effect) ÷ 100), and *m*_*1*_ is the slope of linear regressions from median effect plots using the function

3 $$ log\left( {\frac{fa}{1 - fa}} \right) = f\left( {log\left( {D_{1} x} \right)} \right) $$

The CI-effect graph representing the CI as a function of the associated antibacterial effect was also plotted as well as the log (DRI)-effect plot representing the log of the dose reduction index (DRI) as a function of the associated antibacterial effect. The DRI is the ratio of the concentration of a treatment resulting in an effect *x* alone (D_1_*x*) to the concentration of the same treatment resulting in an effect *x* in combination (D_1_):

4 $$ DRI = \frac{{D_{1} x}}{{D_{1} }} $$

*Ehrlichia canis* suspensions were standardized at 800 cells/well with a 70% infection rate using 96-well culture plates. Solutions of the tested products were used in concentrations determined from their respective IC_50_ The protocol used to determine the antimicrobial effect of the essential oil and doxycycline were an adaptation from Rolain et al. (1998) and Rolain et al. (2002) regarding serial dilutions 0.0625 0.25, 0.5 and 1 times the respective IC_50_ value that was determined for each treatment. Initially, we added 200 μL of medium into each well of a sterile microplate. Subsequently, 50 μL of each product tested in serial dilutions were arranged in an orderly fashion so that we were able to evaluate activity according to the decrease of the essential oil and the synthetic drug. From top to bottom, there is decrease of the essential oil IC_50_, and horizontally from the right to the left there is a decrease of the synthetic drug (Nightingale et al. 2007).

Our results show that in each well there is a unique combination of concentrations between the two substances (i.e., essential oil of *A. conyzoides* and doxycycline).

### Statistical analysis

The analyses were performed using the software GraphPad Prism 5.0 (GraphPad Software, La Jolla, California, USA). The Student’s test was for toxicity analysis and the analysis of variance (ANOVA) was used to obtain data on the rate of inhibition of the microorganisms and the treatment time of the groups. Statistically, significant differences were found with values of p < 0.05. The IC_50_ value was also acquired by linear regression using the software GraphPad Prism 5.0 (GraphPad Software, La Jolla California USA). The effects of the interaction between the treatments and doxycycline were also evaluated by the analysis of the combination of multiple drugs using the software CompuSyn^®^ (Chou and Talalay 1984).

## Results

### Biological assay

**Fig. 1:**
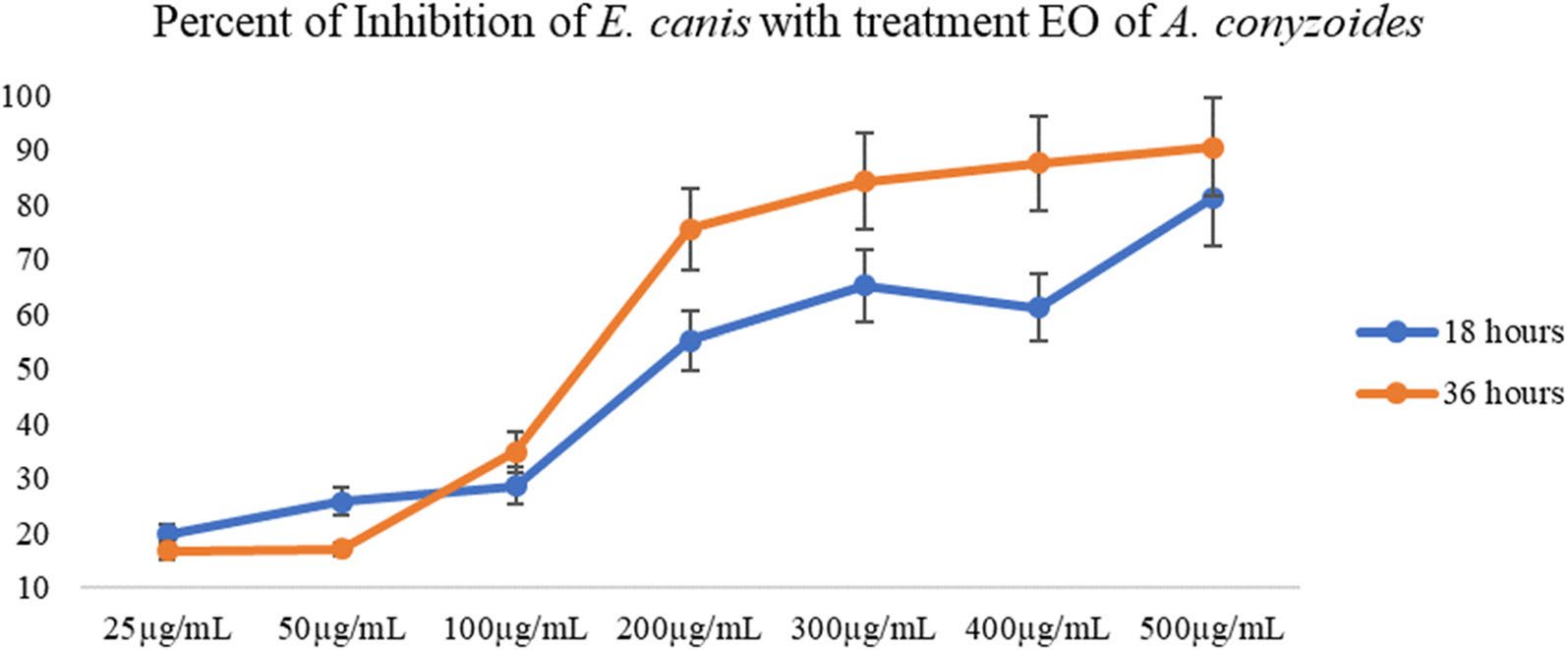
Percent of inhibition of *Ehrlichia canis* in DH82 cells, after 18 and 36 h of treatment as essential oil of *A. conyzoides*, in different concentrations. Each line represents medium and deviant pad of three independent trials

To the authors’ knowledge, this is the first alternative treatment (alternative therapy) based on bioactive compounds with antibacterial activity against *E. canis*. Figure 1 shows the percentage of inhibition of *E. canis* infection in cells after 18 h and 36 h of treatment with the essential oil of *A. conyzoides*. It is noted that at the concentration of 200 µg/mL at 36 h this hydrophobic liquid inhibited a percentage greater than 50% of *E. canis* morulae formation. This result demonstrates the in vitro efficacy of this oil.

### Cell viability

**Fig. 2:**
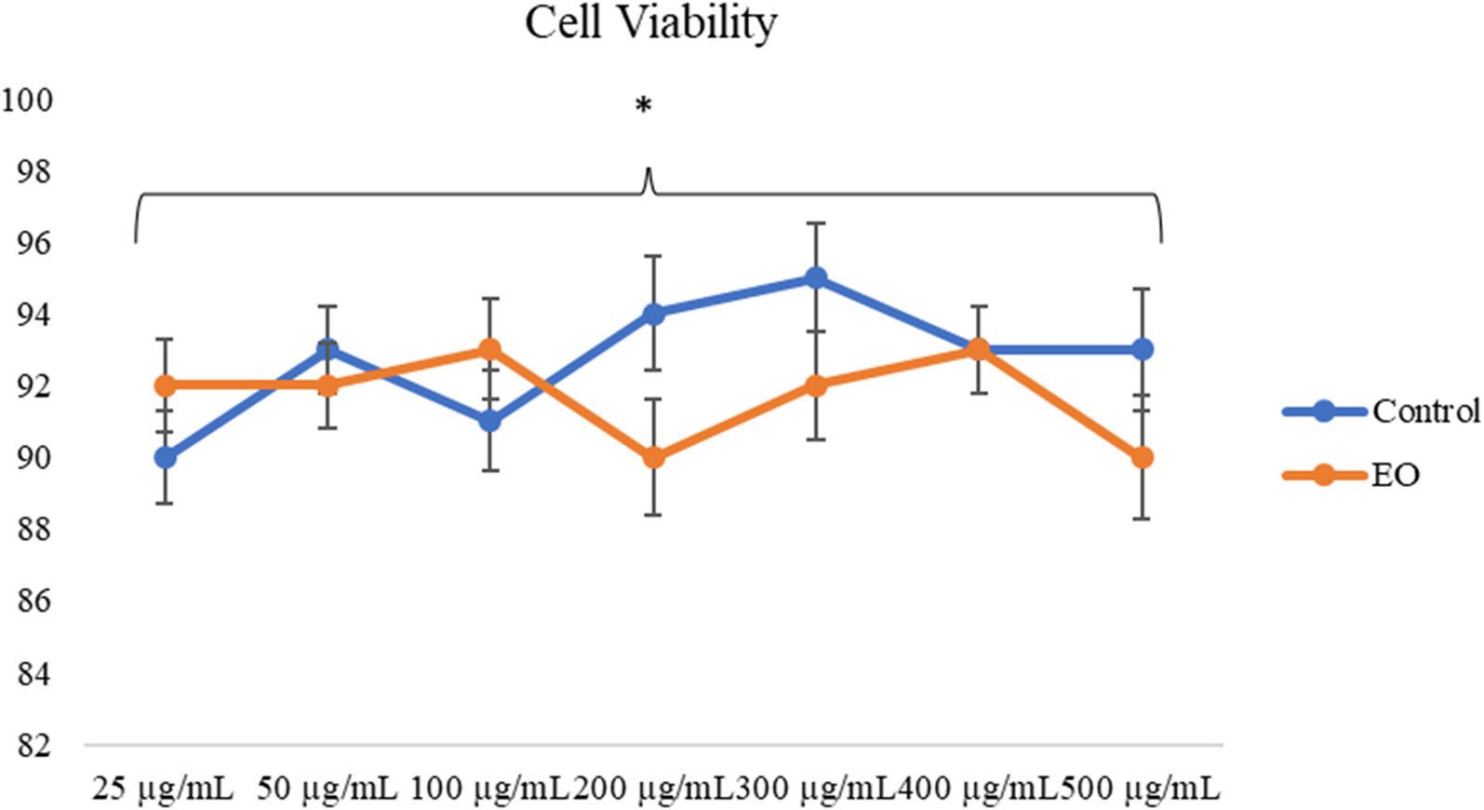
Each row represents mean and standard deviation of three independent assays. Where *p < 0.05 demonstrated that there was no statistically significant difference in relation to the control group according to the unpaired Student’s *t* test with a 95% confidence index

The cytotoxicity of the treatments tested was demonstrated on DH82 cells after 24 h of incubation, and the viability was determined by the trypan blue dye exclusion test. None of the treatments showed cytotoxicity up to a maximum concentration of 500 µg/mL (Fig. 2).

### Association of treatments

The possible interactions between doxycycline and the essential oil of *A. conyzoides* L. were assessed in vitro using the isobologram of non-fixed proportions modified using *E. canis*-infected DH82 cells after 24 h of incubation with the respective treatments tested.

Since doxycycline is the treatment of choice for treating canine ehrlichiosis, we performed the tests of association with the essential oil obtained from *A. conyzoides* to verify if at lower concentrations both treatments could be used to inhibit the percentage of infection in vitro by *E. canis*. We used the Chou and Talalay method to design the hypotheses and evaluate the various combinations (Chou and Talalay 1984; Chou 2010).

**Fig. 3:**
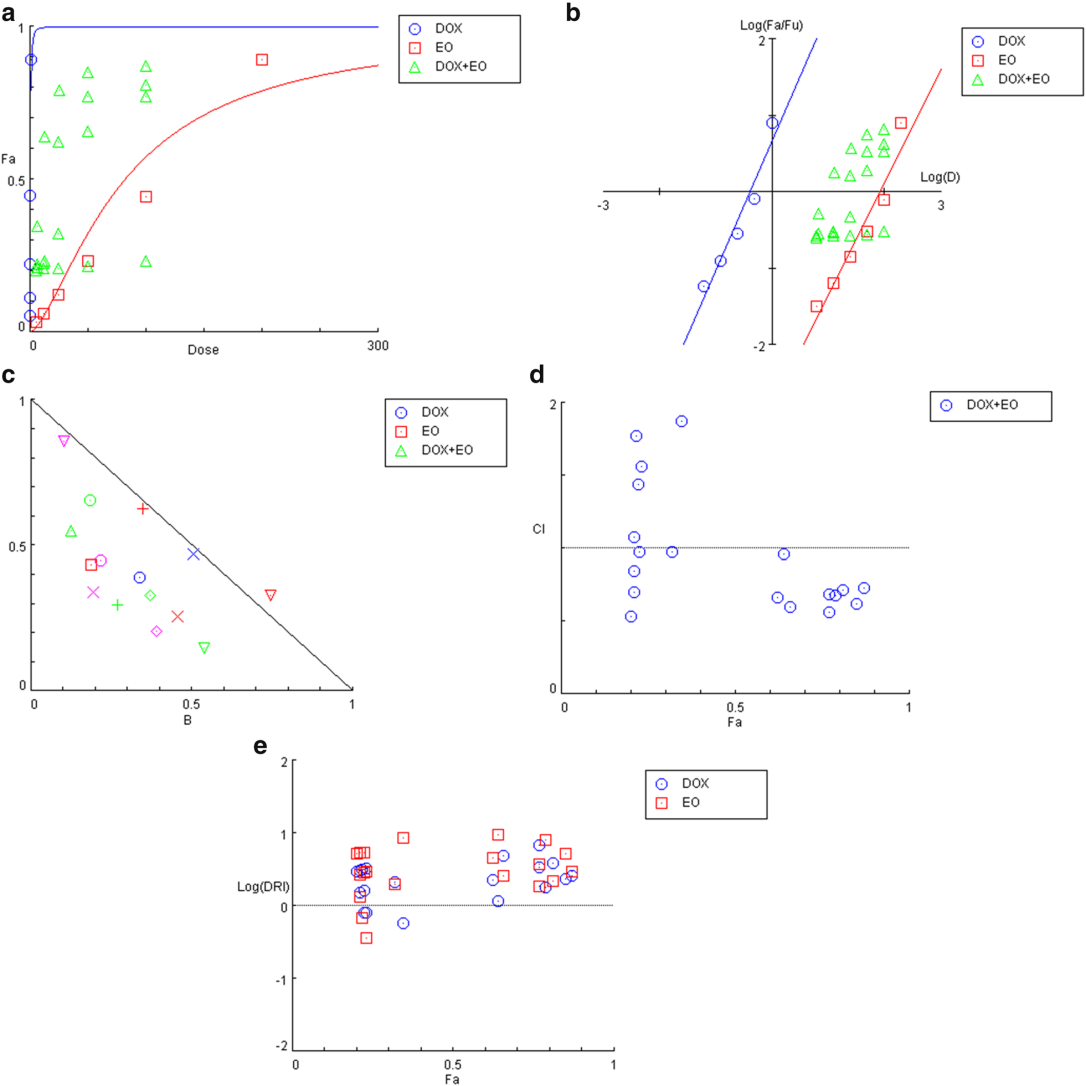
Evaluation of the synergism of doxycycline in combination with essential oil (EO) of *A. conyzoides*. Data sets in blue, red and green correspond to doxycycline, EO, combinations, respectively. **a** Dose–response curves of each individual treatment. Doxycycline was evaluated twice, for each of the combinations with EO. The antibacterial effect was determined by measurement of foci size. **b** Median-effect representation of the dose–response curves for each individual compound, using Eq. (3). fa is the “fraction affected”, or (% effect) ÷ 100. **c** Normalised isobologram that represents, for each combination, the normalised dose of each treatment individually required to reach the observed effect in combination (Eq. 2). **d** CI-effect plot representing the combination index CI, calculated using Eq. (1), of each combination as a function of their associated antibacterial effect. **e** Log (DRI)-effect plot representing the drug reduction index (DRI) of compounds as a function of their antibacterial effect in combination. The DRI is calculated for each drug in each combination according to Eq. (4) and represents the dilution factor required for a drug to reach the same level of inhibition individually compared with it when in combination. The results are representative of 3 independent experiments

The treatments were tested in constant proportions of equipotent concentrations, ranging from 0.0625 to 1 times the respective IC_50_ value that was determined for each treatment (Fig. 3a). The slope m was also determined from the linear regression of the median effect of the plots (Eq. 3), as they reflected the sigmoidicity of the dose–response curves and were used for the calculation of normalized concentrations and treatment reduction indices (Fig. 3b).

Standard isobologram is a graphical way of visualizing synergistic combinations with respect to concentrations. Since D1 is the concentration of treatment 1 responsible for an x effect in combination, and D_1_x the concentration of treatment 1 responsible for an x effect alone, a normalized concentration $\frac{D1}{D1x}$, calculated using Eq. (2), tends to zero as smaller concentrations of the treatments in combination are required to achieve an x effect.

As shown in Fig. 3c, virtually all data points are located in the region where combinations have a synergistic effect, suggesting that the essential oil obtained from *A. conyzoides* acts in synergy with doxycyclines in the in vitro treatment of infected DH82 cells *E. canis*. The IC effect of the graph also allows the visualization of combination effects, based on the combined CI index calculated using Eq. (1). The CI value is represented by a function of the antibacterial effect associated with each combination (Fig. 3d).

Similarly to Fig. 3c, the combination sites that were less than 1 indicate a synergistic effect between the essential oil obtained from *A. conyzoides* and doxycycline. The fact that the compounds show synergism means that their concentration in combination produces an effect that is stronger than when the treatments are used individually in a similar or greater concentration. For this synergistic property the inhibitors can be evaluated by calculating the drug dose reduction index (DRI, Eq. 4) for each treatment of each combination, and is plotted with the log (DRI) (Fig. 3e).

In our case the essential oil obtained from *A. conyzoides* and doxycycline make up combinations that inhibit *E. canis* infection in vitro, the effect becomes stronger, the greater the DRI. Although this result is expected, it does not translate into synergy. DRI is calculated for individual drugs in a given combination effect.

Based on the DRI effect plot, 100% inhibition of infection is achieved when the concentration of a compound in combination can be reduced tenfold compared to the concentration required to achieve the same effect individually.

### EO analysis

Analysis of the essential oil was performed by gas chromatography/mass spectrometry (GCMS) using a Shimadzu^®^ equipment from GCMS-QP2010s; 8 compounds were detected in this oil, and we were able to identify 99.63% of these compounds. The composition of the oil is presented in Table 1.

**Table 1:** Compounds found in the essential oil of *Ageratum conyzoides* L.

Compounds	RT	RI cal.	RI lit.	Area %
Camphene	5970	949	953	0.36
ɑ-Carene	7420	1003	1001	0.22
Bornyl acetate	18,675	1289	1287	0.25
Caryophyllene	24,190	1420	1417	5.1
Humulene	25,575	1455	1452	0.27
Precocene I	26,190	1470	1461	92.75
Bicyclogermacrene	27,340	1498	1500	0.29
Beta-sesquiphellandrene	28,395	1525	1521	0.39
Total identified (%)				99.63

*TR* retention time, *RI cal* retention index calculated, *RI lit* retention index literature NIST (2011) and Adams (2012)

The essential oil of *A. conyzoides* has precocene I as its main compound (92.75%) (Table 1) in terms of chemical composition.

## Discussion

The essential oil of *A. conyzoides* presented IC_50_ against *E. canis* at a concentration of 200 µg/mL. This biological activity is due to the hydrophobic properties of essential oils as their compounds provide these oils with the ability to partition into the lipids of the cellular membrane of bacteria and mitochondria, disarranging the structures and making them more permeable (Alviano and Alviano 2009).

In the present study, the amount of phenolic compounds that is present in the composition of *A. conyzoides* may have caused a disturbance in cell membranes. In addition, these compounds may also have disrupted the proton motive force, the electron flow, and the active transport. As a result, there is coagulation of the cellular contents. EOs also appear to act on membrane proteins—enzymes such as ATPases which are surrounded by lipid molecules—suggesting two possible mechanisms: (i) cyclic, lipophilic hydrocarbons that would accumulate in the lipid bilayer distorting lipid–protein interaction, and (ii) direct interaction of the lipophilic compounds with the hydrophobic parts of the proteins. In previous studies, a number of researchers reported that the presence of essential oils also interferes with the repair mechanism necessary for the cellular division of microorganisms as these oils act on bacterial membranes thus promoting their rupture (Farias and Lima 2000).

The main mechanism of action of doxycycline consists of the inhibition of protein synthesis. It acts effectively in the treatment of canine ehrlichiosis. There are some restrictions though. We suggest that possibly the mechanism of action of the essential oil of *A. conyzoides* against this rickettsia consists of similar biochemical interactions. The bacteriostatic activity and/or the bactericidal activity of EOs are mainly exerted by terpenoid compounds. In general, the main compound present in the essential oil is responsible for the biological activity. However, the combination of these substances may increase the activity of the essential oil since the compounds may interact with each other resulting in a synergistic effect (Feitosa-Alcantara et al. 2017).

In the present study, a synergistic action was observed in the evaluation of the effects of the associations between doxycycline and the essential oil of *Ageratum conyzoides* L., resulting in a reduction of up to 4.89 times the IC_50_ of DOX (see Table 2). The confirmation of the synergistic effects of the essential oil was obtained with the construction of the isobolograms type graphs which show a curve close to the axes for the studied specimen. This assessment suggests that *Ageratum conyzoides* L. is a plant with important and significant antimicrobial activity.

**Table 2:** Proportion, effect, combination index (CI), minimum dose and caloric reduction index (DRI) of the associations between doxycycline and the essential oil test treatment of *A. conyzoides* L.

Associated treatment	Proportion	Effect	CI	Dose DOX (µg/mL)	Dose treat. test (µg/mL)	DRI DOX	DRI treat. test
DOX + EO	$$ {\raise0.7ex\hbox{$1$} \!\mathord{\left/ {\vphantom {1 8}}\right.\kern-0pt} \!\lower0.7ex\hbox{$8$}} $$DOX + $$ {\raise0.7ex\hbox{$1$} \!\mathord{\left/ {\vphantom {1 8}}\right.\kern-0pt} \!\lower0.7ex\hbox{$4$}} $$EO	0.65	0.265	0.125	50	4.90	2.55

*DOX* doxycycline, *EO* essential oil

According to González-Lamothe et al. (2009), accumulated secondary metabolism products of plants may potentiate antibacterial activity favoring antibiotics which action is limited by multidrug resistance mechanisms developed by the microorganisms or as “attenuating virulence” adjusting the host immune response to infection.

The benzopyran nucleus found in the EO constituents of *A. conyzoides* constitutes a molecular pattern for antiprotozoal (Narender et al. 2004), antimicrobial, and antiproliferative activities of the plant (Jardosh and Patel 2013), and has antioxidant ability as well (Mladenovic et al. 2011).

Precocene I was the major compound found in our study. It belongs to the group of chromenes. A number of authors have demonstrated the activity of precocene I against *Plasmodium falciparum* (Severino et al. 2009), *Leishmania* spp. (Narender et al. 2004), and *Trypanosoma cruzi* (Batista et al. 2008) which are obligate intracellular parasites.

A number of compounds were found during the chemical characterization of *A. conyzoides* L. by GC/MS. These compounds may act in different manners against the microorganisms, e.g. terpenes such as the caryophyllene which are able to disrupt the bacterial cell membrane as suggested by Sartori (2005). Its mechanism of action may be associated with its lipophilic character. The accumulation of these compounds within the cell membrane of the microorganism, would result in loss of energy by these cells (Duarte et al. 2011). Greay and Hammer (2015) claim that monoterpenes interfere with the integrity and function of the cell membrane of bacteria by changes in membrane potential, loss of cytoplasmic material, and inhibition of the respiratory chain. In addition, the expression of cytoplasmic and membrane proteins has been implicated in the expression of genes encoding virulence factors (Qiu et al. 2011).

The yield of the essential oil is an important factor that may determine its production in large scale. The yield of the *A. conyzoides* oil collected in São Luís, MA, northeast Brazil was 1.7%. Castro et al. (2004) evaluated 5 different types of accessions of *A. conyzoides* and obtained contents between 0.48% and 0.70%. Other authors including Lima et al. (2014) and Barros et al. (2016) found yields of 0.46% and 0.08%, respectively. This is mainly due to climate, incident solar radiation, relative humidity, and different soil type as previously reported.

The major chemical constituent found in the essential oil of *A. conyzoides* was precocene I with a concentration of 92.75% as reported by Furtado et al. (2005). These authors also found a 62.6% majority in this plant. Martins et al. (2005) evaluated the chemical composition of mentrasto oil and found 38 compounds including precocene I which concentration was 34.4%. Different sesquiterpene hydrocarbons are present in the concentration of 6.05%, and 5.1% correspond to the caryophyllene, which is he main oxygenated sesquiterpene found in the plant.

These variations are due to the fact that the composition of the essential oils are quite complex and generally encompass (involves) various types of compounds. Thus, the standardization of harvest times, the part of the plant collected, and the cultivation under the same environmental conditions help in the identification of varieties that present differences in their chemical composition and concentration. Moreover, factors such as temperature, humidity, and soil may also influence mainly in plant species that have histological structures of essential oil storage on the surface of the leaves (Salgado et al. 2003).

Due to the geographic distribution of the tick vector, *Ehrlichia* infection is highly prevalent in tropical and subtropical regions including Brazil (Andereg and Passos 1999; Aguiar et al. 2013). The need for studies that seek an alternative treatment for this disease has increased over the years due to the long period of treatment required, the toxicity presented by the treatment, and cases of resistance of the rickettsial organism to doxycycline (Andereg and Passos 1999).

In view of the above, the potential of the essential oil of *A. conyzoides* in the inhibition of *E. canis* growth in infected DH82 cells as well as its synergistic effect in the combined treatment with doxycycline which significantly reduced its concentration in the fight against infection are clearly promising bioproducts which may be reproduced industrially.
